# mRNA expression levels and genetic status of genes involved in the EGFR and NF-κB pathways in metastatic non-small-cell lung cancer patients

**DOI:** 10.1186/1479-5876-9-163

**Published:** 2011-09-27

**Authors:** Mariacarmela Santarpia, Ignacio Magri, Maria Sanchez-Ronco, Carlota Costa, Miguel Angel Molina-Vila, Ana Gimenez-Capitan, Jordi Bertran-Alamillo, Clara Mayo, Susana Benlloch, Santiago Viteri, Amaya Gasco, Nuria Mederos, Enric Carcereny, Miquel Taron, Rafael Rosell

**Affiliations:** 1 Pangaea Biotech, USP Dexeus University Institute, Sabino Arana 5- 19, Barcelona, 08028, Spain; 2 Medical Oncology Department, University of Messina, Via Consolare Valeria, Messina, 98125, Italy; 3 Department of Health and Sociomedical Sciences, School of Medicine, University of Alcala de Henares, Crtra. Madrid-Barcelona, km. 33,600, Madrid, 28801, Spain; 4 Catalan Institute of Oncology, Hospital Germans Trias i Pujol, Ctra Canyet s/n, Badalona, 08916, Spain

## Abstract

**Background:**

Metastatic non-small-cell lung cancer (NSCLC) has a dismal prognosis. EGFR is overexpressed or mutated in a large proportion of cases. Downstream components of the EGFR pathway and crosstalk with the NF-κB pathway have not been examined at the clinical level. We explored the prognostic significance of the mRNA expression of nine genes in the EGFR and NF-κB pathways and of BRCA1 and RAP80 in patients in whom EGFR and K-ras gene status had previously been determined. In addition, NFKBIA and DUSP22 gene status was also determined.

**Methods:**

mRNA expression of the eleven genes was determined by QPCR in 60 metastatic NSCLC patients and in nine lung cancer cell lines. Exon 3 of NFKBIA and exon 6 of DUSP22 were analyzed by direct sequencing. Results were correlated with outcome to platinum-based chemotherapy in patients with wild-type EGFR and to erlotinib in those with EGFR mutations.

**Results:**

BRCA1 mRNA expression was correlated with EZH2, AEG-1, Musashi-2, CYLD and TRAF6 expression. In patients with low levels of both BRCA1 and AEG-1, PFS was 13.02 months, compared to 5.4 months in those with high levels of both genes and 7.7 months for those with other combinations (*P *= 0.025). The multivariate analysis for PFS confirmed the prognostic role of high BRCA1/AEG-1 expression (HR, 3.1; *P *= 0.01). Neither NFKBIA nor DUSP22 mutations were found in any of the tumour samples or cell lines.

**Conclusions:**

The present study provides a better understanding of the behaviour of metastatic NSCLC and identifies the combination of BRCA1 and AEG-1 expression as a potential prognostic model.

## Background

**Figure 1:**
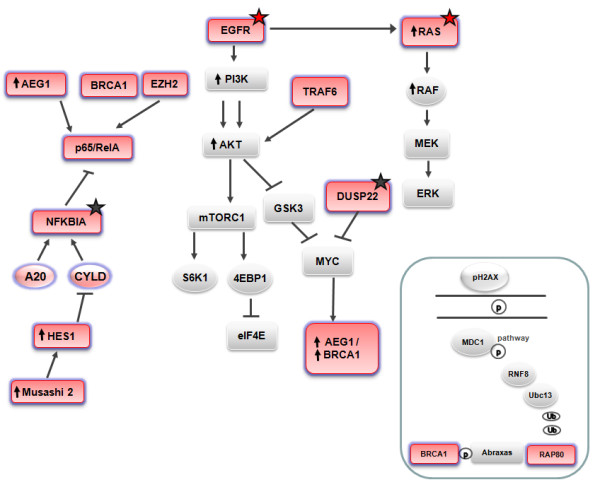
**Inter-relationship and crosstalk among genes**. The eleven genes analyzed in the present study are shown in pink. Red stars indicate mutations that were examined as part of routine clinical practice. Black stars indicate potential mutations that were examined as part of the present study.

Metastatic non-small-cell lung cancer (NSCLC) is currently considered an incurable disease; median overall survival is 12 months with platinum-based chemotherapy [1,2] and only 3.5% of patients survive five years after diagnosis [3]. Therapies targeting EGFR mutations have revolutionized the treatment of NSCLC; however, additional targeted therapies are lacking. More than half of NSCLCs have excessive activation of the epidermal growth factor receptor (EGFR) signaling pathway due to gene amplification or EGFR mutations [4,5]. The activated EGFR receptor may phosphorylate a wide array of intracellular signaling cascades, such as the RAS-RAF-MEK-ERK and the phosphatidylinositol 3-kinase (PI3K)-AKT pathways [3] (Figure 1). Nuclear factor kappa B (NF-κB) is a transcription factor activated by the EGFR pathway [6]. NF-κB inhibitor alpha (NFKBIA), a gatekeeper for EGFR signaling that represses NF-κB, is a major downstream node in the NF-κB and EGFR pathways [6] (Figure 1). We recently observed that increased NFKBIA expression predicted improved progression-free (PFS) and overall survival in EGFR-mutant NSCLC patients treated with erlotinib [7]. However, the functional and clinical impact of crosstalk between the multiple pathways radiating from growth factor receptors remains obscure [8]. The present study sought to elucidate the influence of the genetic status and expression of several genes involved in the NF-κB and EGFR pathways in metastatic NSCLC patients treated with platinum-based chemotherapy (Figure 1).

In lung cancer cells with mutated K-ras, NF-κB is activated by the non-canonical TBK1/IκB kinase (IKK) interaction [9]; blocking IKK activity reduced tumor growth in a mouse lung adenocarcinoma model [10]. In T cell leukemia, the Notch/Hes1 pathway sustains NF-κB activation through repression of cylindromatosis tumor suppressor (CYLD) [11]. CYLD and A20 negatively regulate the NF-κB pathway [12] (Figure 1). High-throughput DNA sequencing analysis of a cancer cell genome of a lung adenocarcinoma patient revealed somatic mutations in K-ras, NFKBIA and DUSP22. DUSP22 is a negative regulator of p38. Active p38 signal transduction plus loss of NFKBIA could lead to aberrant activation of transcription factors via MYC and NF-κB [13] (Figure 1). Outlier expression of Musashi-2 was identified in acute and chronic myeloid leukemias and correlated with shorter survival [14,15]. Musashi-2 inhibits translation of Numb mRNA, which inhibits the Notch pathway, and a significant inverse correlation between Hes1 mRNA levels and Numb status has been observed in NSCLC [16] (Figure 1). Enhancer of zeste homolog 2 (EZH2) activates Ras and p65/RelA (a measure of NF-κB activity) [17], and high EZH2 expression has been correlated with poor prognosis in several tumors, including gastric cancer [18]. Tumor necrosis factor receptor-associated factor 6 (TRAF6) facilitates AKT membrane recruitment and subsequent AKT phosphorylation and activation [19]. TRAF6 influences innate immune response and apoptosis by regulating Toll-like receptor and transforming growth factor-β (TGF-β) signaling, which are involved in NF-κB activation and p38 activation, respectively [20]. BRCA1 also interacts with AKT and promotes its ubiquitination and degradation [21].

Intriguingly, MYC induces expression of BRCA1 [22] and also of astrocyte elevated gene-1 (AEG-1) [23] (Figure 1). Ha-ras activates the PI3K signaling cascade, resulting in increased AEG-1 expression, and AEG-1 in turn activates the NF-κB pathway that regulates expression of genes involved in migration and invasion. The AEG-1-activated PI3K-AKT pathway inhibits apoptosis through phosphorylation of anti-apoptotic AKT substrates [23]. RAP80 is required for optimal accumulation of BRCA1 on damaged DNA foci in response to ionizing radiation. The RAP80/Abraxas complex facilitates the recruitment of BRCA1 to DNA-damaged sites [24] (Figure 1). In a BRCA1-customized study in metastatic NSCLC, PFS and overall survival was influenced by RAP80 expression; in the most favorable subgroup of patients - those with low levels of both BRCA1 and RAP80 - PFS was 14 months [25].

In order to shed light on the clinical impact of the multiple interconnections and crosstalk between these components of the NF-κB and the EGFR pathways, we examined the expression of eleven genes (Figure 1) and the mutational status of two genes (Figure 1) and correlated our findings with outcomes in metastatic NSCLC patients in whom EGFR and K-ras genetic status had previously been determined.

## Methods

### Study population

A total of 60 metastatic NSCLC patients who were visited at the Medical Oncology Service of the USP Dexeus University Institute (Barcelona, Spain) were assessed for mRNA expression of eleven genes (CYLD, A20, EZH2, AEG-1, TRAF6, NFKBIA, p65/RelA, Musashi-2, Hes1, BRCA1, RAP80) and mutational status of NFKBIA and DUSP22 (Figure 1). EGFR and K-ras mutational status had previously been determined in all 60 patients as part of routine clinical practice. Patients were predominantly males; 39 patients had adenocarcinoma, 13 squamous cell carcinoma and 8 large cell carcinoma. Fifty-two patients received first-line platinum-based chemotherapy, seven patients - all with EGFR mutations - received first-line erlotinib, and one received chemotherapy plus erlotinib. Table 1 displays patient characteristics, including the number of chemotherapy lines, the metastatic sites and EGFR and K-ras mutations. All patients provided written informed consent. Approval was obtained from the institutional review board and the ethics committee.

**Table 1:** Characteristics of metastatic NSCLC patients

	N	(%)
Age (yrs)	58 years (range 29-76)	100

Gender		
Male	36	60
Female	24	40

Performance status		
0	14	23.3
1	41	68.3
2	5	8.3

Histology		
Large-cell carcinoma	8	13.3
Adenocarcinoma	39	65
Squamous cell carcinoma	13	21.7

Smoking history		
Current smoker	22	36.7
Never smoked	7	11.7
Former smoker	20	33.3
Unknown	11	18.3

Number of chemotherapy lines		
1	18	30
≥2	42	70

Metastatic site		
Lung	25	41.7
Bone	23	38.3
Brain	10	10
Liver	4	6.7
Pleura	8	13.3
Adrenal	8	13.3
Skin	1	1,7
Others	4	6.7

EGFR mutations (51 patients screened)		
del 19	7	13.7
L858R	2	3.9
Total	9	17.6

K-ras mutations (56 patients screened)	10	17.8

First-line therapy		
Chemotherapy	52	
Erlotinib	7	
Chemotherapy + erlotinib	1	

Response		
Complete response	5	8.9
Partial response	28	50
Stable disease	5	8.9
Progressive disease	13	23.2
Not measurable	9	8.9

### Cell culture and viability

CYLD, A20, EZH2, AEG-1, TRAF6, NFKBIA, p65/RelA, Musashi-2, Hes1, BRCA1 and RAP80 mRNA expression levels were also analyzed in nine lung cancer cell lines: four K-ras-mutated cell lines (A549, NCI-H23, H460, Calu-6); two EGFR-mutated cell lines (PC9, H1975); and three K-ras-and EGFR-wild type cell lines (NCI-H510, SK-MES-1, HCC-827WT).

All tissue culture materials were obtained from Biological Industries (Kibbutz Beit Haemek, Israel) or Invitrogen (Paisley, Scotland, UK). H460, Calu-6, A549, H23, H1975 and SK-MES-1 human lung tumor cell lines were provided by the American Type Culture Collection. The H510 and HCC-827WT cell line were provided by the University of Pamplona; HCC-827WT was derived from HCC-827 but lost the original EGFR exon 19 deletion upon prolonged culture. PC-9 was provided by Roche Inc. (Basel, Switzerland) with the authorization of Dr. Mayumi Ono. All cell lines were maintained in RPMI medium supplemented with 10% FBS, 50 μg/mL penicillin-streptomycin and 2 mM L-Glutamine. All cells were grown in a humidified atmosphere with 5% CO2 at 37°C.

Cell viability was assessed by the Thiazolyl Blue Tetrazolium Bromide (MTT) (Sigma, St. Louis, MO) assay. Cells from each cell line were seeded at 8000 to 10000 per well (except for H209, where 50000 cells were used) in 96-well plates. The concentration of drug required for 50% growth inhibition (IC_50_) for cisplatin and erlotinib upon 24 h exposure was assessed by standard procedures. After treatment, cells were incubated with medium containing MTT (0.75 mg/mL in medium) for 1-2 h at 37°C. Culture medium with MTT was removed and formazan crystals reabsorbed in 100 μL DMSO (Sigma, St. Louis, MO). Cell viability was determined by measuring the absorbance at 590 nm, using a microplate reader (BioWhittaker, Walkersville, MD).

### Microdissection

All specimens were formalin-fixed, paraffin-embedded tumor tissues (FFPET) and were stained with haematoxilin/eosin and assessed by the pathologist of the Laboratory of Molecular Biology of the USP Dexeus University Institute (Barcelona, Spain). Microdissection was then performed as previously described [26].

### Gene expression

Gene expression profiling was performed on RNA isolated from the tumor tissue specimens. RNA extraction, retrotranscription analysis and real-time PCR were performed as previously described [26]. Primers and probes for gene expression characterization of β-actin, CYLD, A20, AEG-1, TRAF6, NFKBIA, p65/RelA, Musashi-2, Hes1, BRCA1, and RAP80 were designed according to their Ref Seq in http://www.ncbi.nlm.nih.gov/sites/entrez?db=gene (Additional File 1, Table S1). EZH2 gene expression was analyzed with the Hs01016789_m1 assay from Applied Biosystems (AB; Foster City, CA, USA).

### Gene mutations

EGFR, K-ras, NFKBIA and DUSP22 mutations were assessed. Tumor cells were resuspended in 20 μL of PCR buffer (Ecogen, Barcelona, Spain) plus proteinase K and incubated from 4 hours to overnight at 60°C. Proteinase was inactivated at 95°C for 10 min, and the cell extract submitted to PCRs.

EGFR mutations in exons 19 and 21 were determined as previously described [27]. Mutations in codons 12 and 13 of K-ras were analyzed by a single round of PCR followed by sequencing. Primers for exon 2 of K-ras were designed according to its Ref Seq in http://www.ensembl.org/index.html, using the www primer tool (http://biotools.umassmed.edu/bioapps/primer3_www.cgi). Primers were as follows: forward 5'-ACATGTTCTAATATAGTCACATTTTCA-3', and reverse 5'-GGTCCTGCACCAGTAATATGCA-3'. PCR was performed in 25-μL volumes adding 3 μL of sample, 1 U of HotStart Taq Polymerase (Qiagen, Hilden, Germany), 2.5 μL of PCR buffer x10, 250 μM dNTPs, 3.5 mM MgCl_2 _and 0.4 pM of each primer. Amplification was as follows: 45 cycles of 30 sec at 95°C, 30 sec at 51°C and one min at 72°C. PCR products were visualized on a 2% agarose gel. Sequencing was performed by standard procedures using forward and reverse nested primers with the ABI Prism 3100 DNA Analyzer (AB).

In lung tumor cell lines and in 30 NSCLC patients with sufficient tumor DNA, somatic mutations in exon 3 of NFKBIA and exon 6 of DUSP22 were also analyzed. Primers for NFKBIA and DUSP22 were designed according to their Ref Seq in http://www.ensembl.org/index.html, using the www primer tool (http://biotools.umassmed.edu/bioapps/primer3_www.cgi) and flanking the mutational sites previously reported [13]. Primers were as follows: DUSP22 forward 5'-TCTGAAACTGCCCTCACACA-3', and reverse 5'-TGCATCTCTGATGTCCCCTA -3'; NFKBIA forward 5'-TCTGGTCTCTCTTGCATTCG-3', reverse 1 5'-GGCAGGGAGGCAGACATAC-3' and reverse 2 (for PCR sequencing) 5'-GGCAGACATACCATTGT-3'. PCR was performed in 25-μL volumes adding 3 μL of sample, 1 U of HotStart Taq Polimerase (Qiagen), 2.5 μL of PCR buffer x10, 250 μM dNTPs, 1.5 (NFKBIA) or 3.5 mM MgCl_2 _(DUSP22) and 0.4 pM of each primer. Amplification was as follows: 45 cycles of 30 sec at 95°C, 30 sec at 54°C (DUSP22) or 57°C (NFKBIA) and one min at 72°C. PCR products were visualized on a 2% agarose gel. Sequencing was performed by standard procedures using forward and reverse nested primers with the ABI Prism 3100 DNA Analyzer (AB).

### Statistical analyses

This was a retrospective analysis exploring whether the altered expression of genes involved in the EGFR and NF-κB pathways correlated with clinical features and outcome in NSCLC. Gene expression levels were examined as continuous variables or dichotomized at the median value.

All efficacy results were assessed in all 60 patients. Objective responses were recorded according to the RECIST criteria. Patients achieving a complete or partial response were considered "responders", and all other patients were considered "non-responders". PFS was calculated from the time of diagnosis of metastatic disease until radiographic progression or death. Median overall survival was calculated from the time of diagnosis of metastatic disease until death or loss to follow-up or last available date. Survival curves were drawn with the Kaplan-Meier method and compared with a two-sided log-rank test.

In order to asses correlation between clinical and genetic characteristics, the Fisher exact test was used for 2-by-2 tables and the Chi-square test in tables of higher order when categorical variables were compared, while ANOVA or Kruskall-Wallis was used to assess differences of continuous variables. Normality of continuous variables was checked by means of the Kolmogorov-Smirnov test. The Pearson correlation coefficient analysis was used to determine the correlation between different genes.

A univariate Cox regression analysis was used to assess the association between each potential prognostic factor and PFS or overall survival with HRs and their 95% CIs. A multivariate Cox proportional hazards regression model was estimated with gender, age, Eastern Cooperative Oncology Group (ECOG) performance status (PS), histology (adenocarcinoma versus non-adenocarcinoma), smoking status (current smoker versus former smoker and never smoked), EGFR mutations, K-ras mutations, and BRCA1/AEG-1 risk groups as covariates. Stepwise analysis (forward and backward) was used to determine the improvement of the fit. In addition, the number of treatment lines, number of metastatic sites, and the presence of bone or brain metastases were included as covariates to estimate HRs of death.

The level of significance was set at ≤ 0.05. All analyses were performed using Statistical Package for the Social Sciences (SPSS) for Windows version 17.0 (SPSS Inc, Chicago, IL, USA).

## Results

### Gene expression

Gene expression of the eleven genes was successfully analyzed in all cell lines. Significant correlations were found between the expression levels of several genes: BRCA1 correlated significantly with AEG-1 (r^2^: 0.76; *P *= 0.002); RAP80 correlated with A20 (r^2^: 0.83; *P *= 0.001); and NFKBIA correlated with p65/RelA (r^2^: 0.71; *P *= 0.006). A significant correlation was also observed between the presence of K-ras mutations and high AEG-1 and NFKBIA expression (r^2^: 0.99; *P *= 0.001). BRCA1 expression showed a significant correlation with sensitivity to cisplatin (IC_50_) (r^2^: 0.65; *P *= 0.008). No other significant correlation was observed.

Gene expression of the eleven genes was also successfully analyzed in all 60 tumor samples. The median values of gene expression are shown in Additional File 1, Table S2.

Significant correlations were found between several genes; for example, BRCA1 expression correlated significantly with EZH2 (Additional File 1, Figure S1), AEG-1, Musashi-2, CYLD and TRAF6, and AEG-1 expression correlated with NFKBIA, Musashi-2, p65/RelA and TRAF6 (Additional File 1, Table S3). In the lung cancer cell lines, a similar correlation was found, including a strong association between BRCA1 and AEG-1 expression.

A significant correlation was observed between the presence of K-ras mutations and high AEG-1 expression (*P *= 0.04) and high NFKBIA expression (*P *= 0.04) (Additional File 1, Table S4); in the lung cancer cell lines, a similar correlation was found. However, there was no correlation between EGFR mutational status and expression levels of any of the genes analyzed (Additional File 1, Table S5). A correlation was observed between a higher number of metastatic sites and high Hes1 expression levels (*P *= 0.002) (Additional File 1, Table S6). No other correlation between gene expression levels and clinical features, including response rate, was observed.

### PFS and overall survival

With a median follow-up of 17.62 months (range, 2.04-152.17 months), median PFS was 7.43 months (95% confidence interval [CI], 5.76-9.10 months), and median overall survival was 28.16 months (95% CI, 18.98-37.33 months) (Additional File 1, Figure S2A). For the subgroup of 51 patients with wild-type EGFR, who were treated with chemotherapy, median overall survival was 26.45 months (95% CI, 16.57-36.33 months) (Additional File 1, Figure S2B).

**Figure 2:**
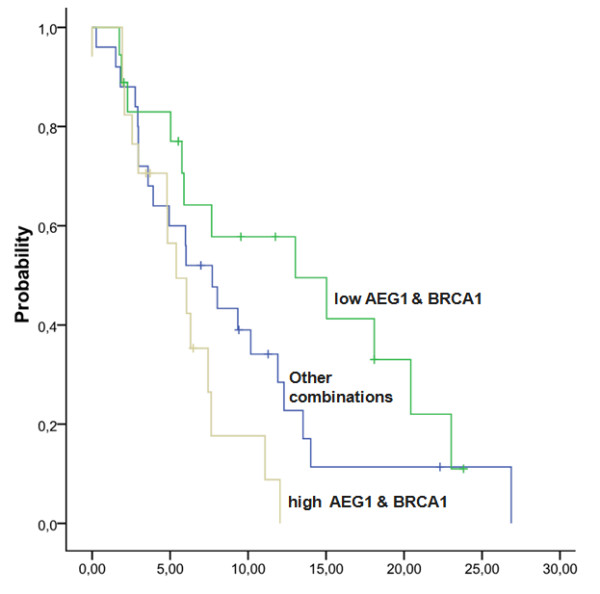
**PFS according to expression levels of BRCA1 and AEG-1**. In patients with low levels of both BRCA1 and AEG-1, PFS was 13.02 months, compared to 5.4 months in those with high levels of both genes and 7.7 months for those with other combinations (*P *= 0.025).

In a univariate analysis for PFS, where only gene expression levels were included, only AEG-1 expression surfaced as a significant prognostic marker (hazard ratio [HR], 1.43; *P *= 0.006) (Additional File 1, Table S7). When AEG-1 expression was analyzed by terciles, patients in the lowest tercile had a PFS of 12.3 months, compared to 9.3 months for those in the intermediate tercile and 4.8 months for those in the highest tercile (*P *= 0.002) (Additional File 1, Figure S3). Based on the correlation observed between BRCA1 and AEG-1 expression in the cell lines and on our previous experience of the role of BRCA1 as a predictive marker of PFS to erlotinib [26], we further investigated the combined effect of BRCA1 and AEG-1 expression on PFS in this series of patients. In patients with low levels of both BRCA1 and AEG-1, PFS was 13.02 months, compared to 5.4 months in those with high levels of both genes and 7.7 months for those with other combinations (*P *= 0.025) (Figure 2). In the final univariate analysis, including all clinical and molecular variables, only this two-gene combination was significantly associated with PFS. The HR for high expression of both BRCA1 and AEG-1 was 3.08 (95% CI, 1.3-7.1; *P *= 0.009) (Table 2). The multivariate analysis for PFS confirmed the prognostic role of high BRCA1/AEG-1 expression (HR, 3.1; 95% CI, 1.3-7.4; *P *= 0.01) (Table 2). Overall survival for the three subgroups was similar, though the differences were not significant (Additional File 1, Figure S4).

**Table 2:** Univariate and multivariate analyses of PFS

Univariate Analysis			
	**N**	**HR (95% CI)**	**p**
Age (years)	60	1.01 (0.98-1.04)	0.63

PS 0	14	1.03 (0.52-2.04)	0.94
PS 1-2	46	1 Ref.	

Male	36	1.48 (0.81-2.70)	0.21
Female	24	1 Ref.	

Adenocarcinoma	39	1 Ref.	0.05
Non-adenocarcinoma	21	1.84 (1.01-3.33)	

Smoker	22	1.26 (0.64-2.49)	0.51
Non- smoker	27	1 Ref.	

EGFR mutated	9	1 Ref.	0.32
EGFR wild-type	41	1.55 (0.65-3.73)	

K-ras mutated	10	1.21 (0.58-2.53)	0.62
K-ras wild-type	46	1 Ref.	

Low BRCA1&AEG1 expression	18	1 Ref.	
High BRCA1&AEG1 expression	17	3.08 (1.33-7.15)	0.009
Other combinations of BRCA1&AEG1 expression	25	1.70 (0.82-3.53)	0.15

**Multivariate Analysis**			

Low BRCA1&AEG1 expression	18	1 Ref.	
High BRCA1&AEG1 expression	17	3.11 (1.30-7.47)	0.01
Other combinations of BRCA1&AEG1 expression	25	2.18 (0.99-4.78)	0.05

Adenocarcinoma	39	1 Ref.	
Non-adenocarcinoma	21	1.52 (0.79-2.91)	0.21

Male	36	1.60 (0.84-3.07)	0.16
Female	24	1 Ref.	

Only histology and the presence of bone metastases were significant factors in both the univariate and multivariate analyses for survival, while in the multivariate analysis, the number of chemotherapy lines was also associated with survival (Table 3).

**Table 3:** Univariate and multivariate analyses of overall survival

Univariate Analysis			
	**N**	**HR (95% CI)**	**p**

Age (years)	60	1.04 (0.99-1.10)	0.16

PS 0	14	0.89 (0.32-2.44)	0.81
PS 1-2	46	1 Ref.	

Male	36	1.09 (0.45-2.61)	
Female	24	1 Ref.	0.85

Adenocarcinoma	39	1 Ref.	
Non-adenocarcinoma	21	2.88 (1.21-6.87)	0.02

Smoker	22	0.96 (0.34-2.68)	
Non-smoker	27	1 Ref.	0.93

EGFR mutated	9	1 Ref.	
EGFR wild-type	41	1.19 (0.38-3.68)	0.77

K-ras mutated	10	0.81 (0.24-2.78)	0.73
K-ras wild-type	46	1 Ref.	

Low BRCA1&AEG1 expression	18	1 Ref.	
High BRCA1&AEG1 expression	17	2.80 (0.84-9.32)	0.10
Other combinations of BRCA1&AEG1 expression	25	1.84 (0.62-5.41)	0.27

1 treatment line	18	1 Ref.	
≥ 2 treatment lines	42	0.44 (0.16-1.22)	0.12

1 metastatic site	30	1 Ref.	
≥ 2 metastatic sites	24	1.46 (0.57-3.72)	0.43

Bone metastases	23	3.06 (1.15-8.16)	0.03
No bone metastases	31	1 Ref.	

Brain metastases	10	1 Ref.	
No brain metastases	44	1.86 (0.54-6.48)	0.33

**Multivariate Analysis**			

Adenocarcinoma	36	1 Ref.	
Non-adenocarcinoma	18	3.17 (1.21-8.31)	0.02

1 treatment line	14	7.22 (1.96-26.64)	0.003
≥ 2 treatment lines	40	1 Ref.	

Bone metastases	23	4.73 (1.58-14.17)	0.005
No bone metastases	31	1 Ref.	

### Gene mutations

EGFR and K-ras mutations were examined as part of routine clinical assessment (Table 1). Somatic mutations in exon 3 of NFKBIA and exon 6 of DUSP22 gene were not found in any of the tumor samples or in any of the cell lines analyzed. Only one patient sample harbored a silent polymorphism (CTC > CTT; Leu), with no amino acid change.

## Discussion

The present study shows that the routine molecular characterization of NSCLC patients is feasible as part of daily clinical practice. The findings on gene expression highlight some of the complex interconnections and crosstalk between different components of the EGFR and NF-κB pathways, which has not been previously explored in lung cancer. BRCA1 mRNA expression was closely related to that of several oncogenes, including EZH2, AEG-1 and Musashi-2 (Additional File 1, Table S3). Interestingly, an integrated 150-gene signature from multiple transgenic models of tumors intrinsic to the functions of the Simian virus 40 T/t antigen was associated with aggressive breast, prostate and lung carcinomas. Both BRCA1 and EZH2 were overexpressed in this gene signature [28], mirroring our findings in the present study (Additional File 1, Figure S1). The T/t-antigen signature was found in all small-cell and squamous cell carcinomas and in a subset of adenocarcinomas [28]. In our previous study in resected NSCLC, we observed that BRCA1 expression was higher in squamous cell than in adenocarcinomas [29]; in addition, in our experience, the expression of BRCA1 and EZH2 was significantly higher in small-cell than in NSCLC. BRCA1 overexpression has been related to poor prognosis in resected NSCLC [29,30] and to shorter PFS to erlotinib in metastatic EGFR-mutant NSCLC patients [26]. Numerous reports also indicate that EZH2 overexpression correlates with poor prognosis in several tumors [17,18,31]. EZH2 activates the Ras and NF-κB pathways [17].

We had previously found that low levels of NFKBIA expression hamper the efficacy of erlotinib in NSCLC patients harboring EGFR mutations. However, in the present study, neither NFKBIA expression nor that of other active components of the NF-κB pathway was associated with outcome. This could be due to the limited number of patients examined. However, AEG-1 expression was associated with outcome. Overexpression of AEG-1 leads to activation of the NF-κB pathway. Although neither NFKBIA nor p65/Rel A was associated with outcome, both were closely correlated with the expression of AEG-1 (Additional File 1, Table S3). We can thus infer that AEG-1 mRNA expression could be a useful biomarker that could be a surrogate of the NF-κB function. AEG-1 is a multifunction oncogene activated via the PI3K-AKT pathway that inhibits apoptosis through the phosphorylation of anti-apoptotic AKT substrates [23]. AEG-1 overexpression correlates with chemoresistance in breast cancer [32]and poor prognosis in NSCLC [33].

In the present study, the median survival of EGFR wild-type patients was 26.45 months, which is higher than the reported median survival of 8-11 months for stage IV chemotherapy-treated NSCLC patients. Overall survival can be influenced by the number of chemotherapy lines after disease progression. In the present study, the majority of patients received more than two lines of treatment for metastatic disease, and the multivariate analysis showed that a higher number of treatment lines was significantly associated with a longer survival (*P *= 0.003). We can speculate that the higher median survival rates may have led to correlations with gene expression that may not extend to other EGFR-wild-type patients; this issue can be clarified in future studies focusing on patients who do not receive more than two lines of treatment. However, in our previous phase II BRCA1-based customized chemotherapy study of NSCLC patients with wild-type EGFR, a subgroup of patients attained a median survival exceeding 26 months [25]. In the majority of patients in the present study, the second- or third-line treatment was also based on BRCA1 mRNA expression levels.

In the present study, low AEG-1 expression was associated with longer PFS, and the combination of low BRCA1 and AEG-1 expression further identified a favorable subgroup of patients in whom PFS was 13 months. In future studies, it could be of great interest to examine BRCA1 and related DNA repair genes in conjunction with AEG-1.

## Conclusions

This study has provided a better understanding of the behavior of metastatic NSCLC and has identified the combination of BRCA1 and AEG-1 expression as a potential model that can determine prognosis to platinum-based chemotherapy in patients with wild-type EGFR and to erlotinib treatment in patients with EGFR mutations. This study is the first of its kind to analyze the multiple genes involved in the NF-κB and EGFR pathways; as such, it has demonstrated the feasibility of performing these analyses in the context of daily clinical practice and has paved the way for further research in this field.

## Competing interests

The authors declare that they have no competing interests.

## Authors' contributions

MS, RR conceived the study, participated in its design and drafted the manuscript. MAM-V, AG-C, JB-A, CM, SB carried out the molecular genetic studies. CC carried out the molecular genetic analyses and helped to draft the manuscript. IM, SV, AG, NM, EC made substantial contributions to acquisition of data. MS, MT, RR made substantial contributions to the analysis and interpretation of data. MS-R performed the statistical analyses. All authors read and approved the final manuscript.

## Supplementary Material

Additional file 1 **supplementary figures and tables**. A pdf file including the following figures and tables: **Figure S1**. Correlation between expression levels of BRCA1 and EZH2. **Figure S2**. Median overall survival for all 60 patients (2A) and for 51 patients with wild-type EGFR treated with chemotherapy (2B). **Figure S3**. PFS according to AEG-1 expression by terciles. **Figure S4**. Overall survival according to levels of BRCA1 and AEG-1 expression (low levels of both genes versus high levels of both genes versus other combinations). **Table S1**. Primers and probes used for each of the genes analyzed. **Table S2**. Median expression values of each of the genes analyzed. **Table S3**. Correlation of the expression levels of the 11 genes analyzed. **Table S4**. Gene expression levels according to the presence or absence of K-ras mutations. **Table S5**. Gene expression levels according to the presence or absence of EGFR mutations (deletion in exon 19 or L858R in exon 21). **Table S6**. Correlation between gene expression levels and number of metastatic sites. **Table S7**. Cox regression model for PFS including only gene expression levels. Click here for file
