# Supplementary material for: mRNA expression levels and genetic status of genes involved in the EGFR and NF-κB pathways in metastatic non-small-cell lung cancer patients
Source: J Transl Med. 2011 Sep 27;9:163. doi: 10.1186/1479-5876-9-163 (PMC3203857; doi:10.1186/1479-5876-9-163)
Supplement: Additional file 1 — supplementary figures and tables. A pdf file including the following figures and tables: Figure S1. Correlation between expression levels of BRCA1 and EZH2. Figure S2. Median overall survival for all 60 patients (2A) and for 51 patients with wild-type EGFR treated with chemotherapy (2B). Figure S3. PFS according to AEG-1 expression by terciles. Figure S4. Overall survival according to levels of BRCA1 and AEG-1 expression (low levels of both genes versus high levels of both genes versus other combinations). Table S1. Primers and probes used for each of the genes analyzed. Table S2. Median expression values of each of the genes analyzed. Table S3. Correlation of the expression levels of the 11 genes analyzed. Table S4. Gene expression levels according to the presence or absence of K-ras mutations. Table S5. Gene expression levels according to the presence or absence of EGFR mutations (deletion in exon 19 or L858R in exon 21). Table S6. Correlation between gene expression levels and number of metastatic sites. Table S7. Cox regression model for PFS including only gene expression levels. [file 1479-5876-9-163-S1.PDF]

mRNA expression levels and genetic status of genes involved in the EGFR and NF-κB pathways in metastatic non-small-cell lung cancer patients

Mariacarmela Santarpia, Ignacio Magri, Maria Sanchez-Ronco, Carlota Costa, Miguel Angel Molina-Vila, Ana Gimenez-Capitan, Jordi Bertran-Alamillo, Clara Mayo, Susana Benlloch, Santiago Viteri, Amaya Gasco, Nuria Mederos, Enric Carcereny, Miquel Taron, Rafael Rosell

ADDITIONAL MATERIAL

The following additional figures and tables are included in this document:

Figure S1. Correlation between expression levels of BRCA1 and EZH2

Figure S2. Median overall survival for all 60 patients (2A) and for 51 patients with wild-type EGFR treated with chemotherapy (2B)

Figure S3. PFS according to AEG-1 expression by terciles

Figure S4. Overall survival according to levels of BRCA1 and AEG-1 expression (low levels of both genes versus high levels of both genes versus other combinations)

Table S1. Primers and probes used for each of the genes analyzed

Table S2. Median expression values of each of the genes analyzed

Table S3. Correlation of the expression levels of the 11 genes analyzed

Table S4. Gene expression levels according to the presence or absence of K-ras mutations

Table S5. Gene expression levels according to the presence or absence of EGFR mutations (deletion in exon 19 or L858R in exon 21)

Table S6. Correlation between gene expression levels and number of metastatic sites

Table S7. Cox regression model for PFS including only gene expression levels

Figure S1. Correlation between expression levels of BRCA1 and EZH2

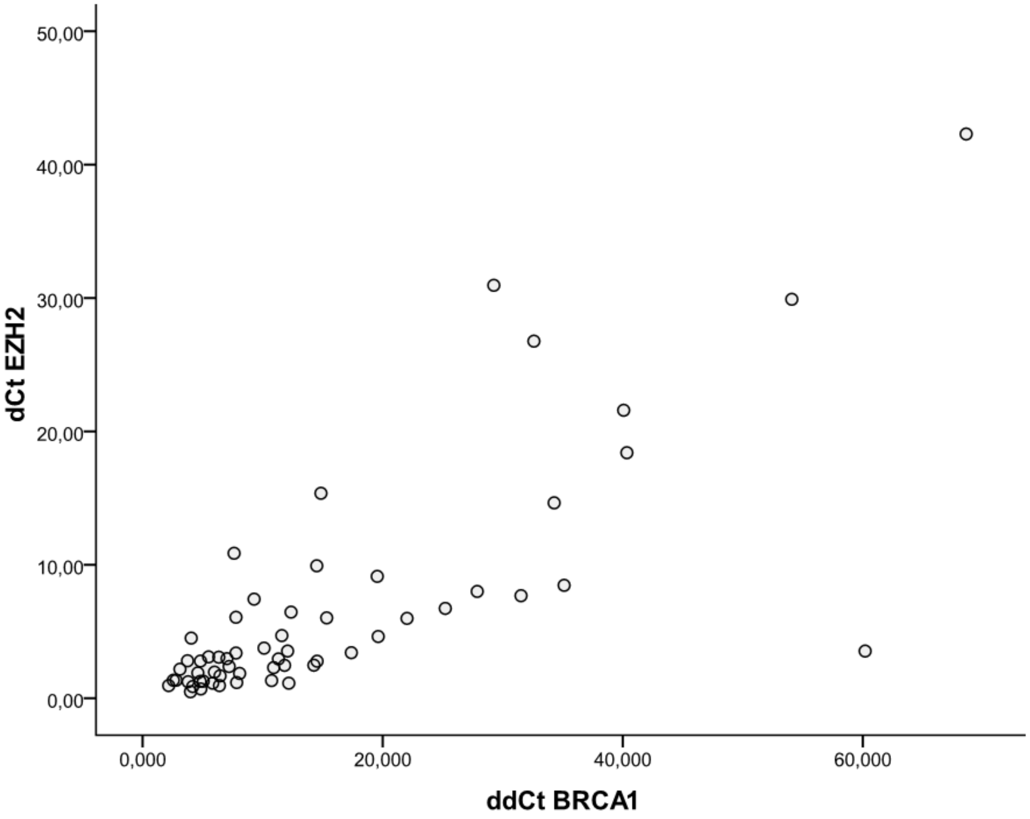

Figure S2. Median overall survival for all 60 patients (2A) and for 51 patients with wild-type EGFR treated with chemotherapy (2B)

2A

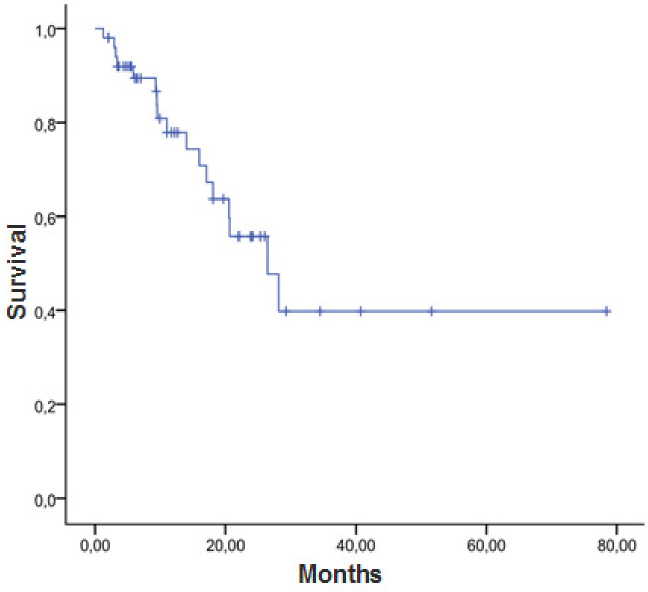

2B

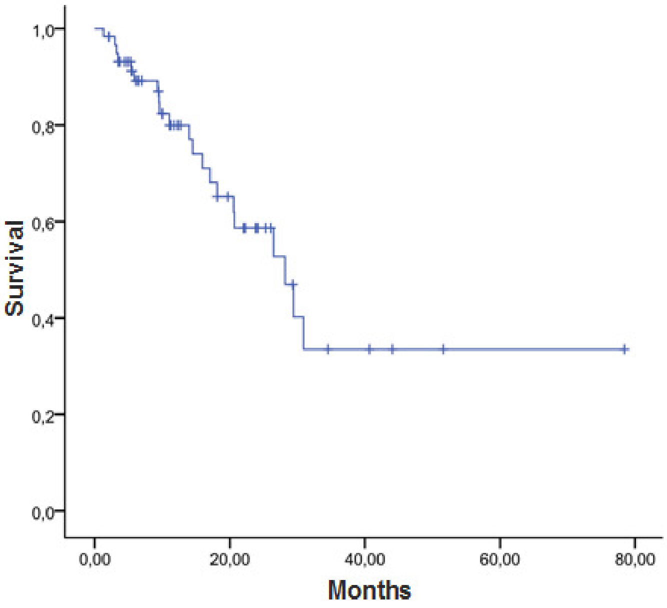

Figure S3. PFS according to AEG-1 expression by terciles

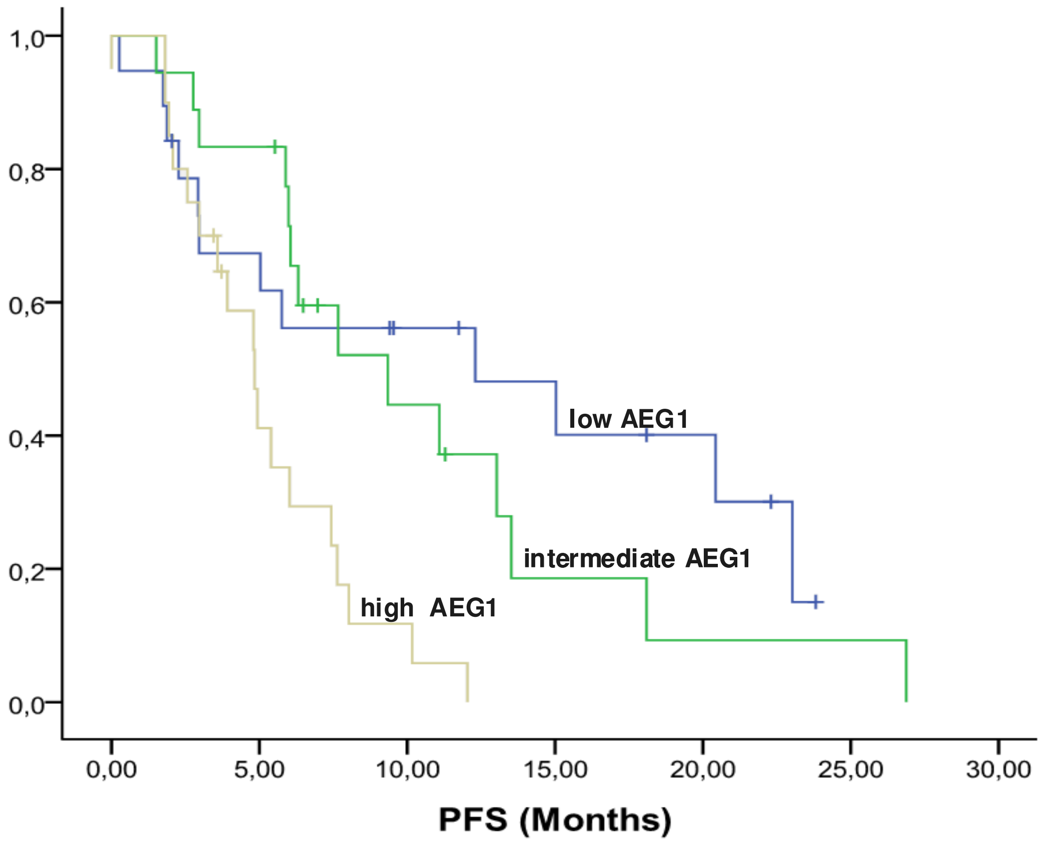

Figure S4. Overall survival according to levels of BRCA1 and AEG-1 expression (low levels of both genes versus high levels of both genes versus other combinations)

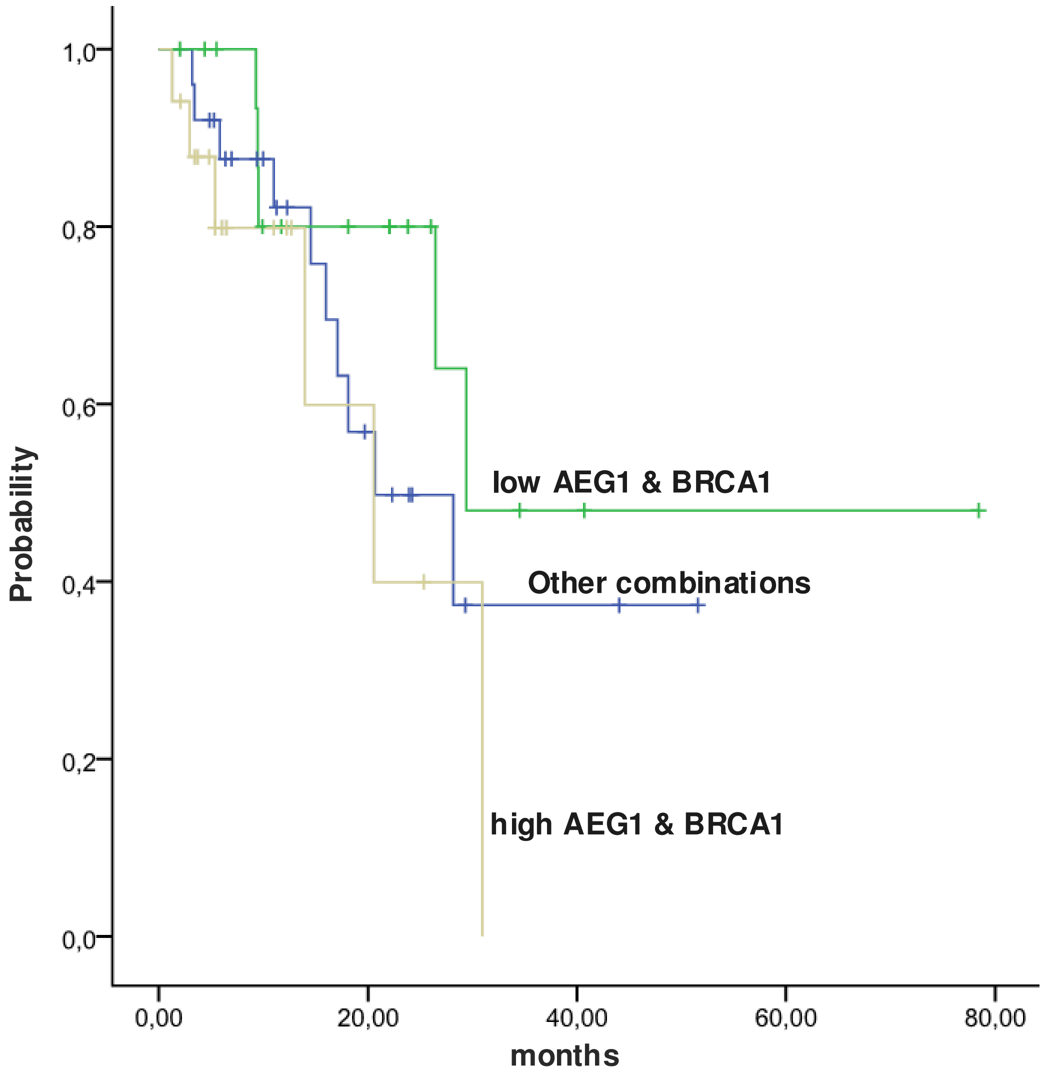

Table S1. Primers and probes used for each of the genes analyzed

Gene	Primers		Probes
**β-ACTIN**	F	5´ TGAGCGCGGCTACAGCTT 3´	6FAM 5´ ACCACCACGGCCGAGCGG 3´ TAMRA
	R	5´ TCCTTAATGTCACGCACGATTT 3´	
**BRCA1**	F	5´GGCTATCCTCTCAGAGTGACATTTTA 3´	6FAM 5´CCACTCAGCAGAGGG 3´ MGB
	R	5´GCTTTATCAGGTTATGTTGCATGGT 3´	
**RAP80**	F	5´ ACATCAAGTCTTCAGAAACAGGAGC 3´	6FAM 5´ TCAGGGTGCCTTCACCA 3´MGB
	R	5´ TGCAGCCTGCCTCTTCCAT 3´	
**AEG-1**	F	5´ GGGGAAGGAGTTGGAGTGAC 3´	6FAM 5´ AATATTTTCTGGCATTGGGTCTA 3´ MGB
	R	5´ GTAGACTGAGAAACTGGCTCAGCAG 3´	
**CYLD**	F	5´ GCTTAGGATATTTTGTTGGTGTGGAC 3´	6FAM 5´ ATGGATAACCCTATTGGCAAC 3´ MGB
	R	5´ CTCCATCAAATCTTCCATCCCA 3´	
**A20**	F	5´ TGGAAAGCCAGAAGAAACTCAACT 3´	6FAM 5´ GGTGTCGAGAAGTC 3´ MGB
	R	5´ CCGTCACCGTTCGTTTTCA 3´	
**TRAF6**	F	5´ TTGTTTGCACAAGATGGAACTGA 3´	6FAM 5´ TTGAGGATCATCAAGCACATT 3´ MGB
	R	5´ TGGGGACAATCCATAAGAGCA 3´	
**NFKBIA**	F	5´ TCCTCAACTTCCAGAACAACCTG 3´	6FAM 5´ CAGCAGACTCCACTCC 3´ MGB
	R	5´ TTCTGGCTGGTTGGTGATCA 3´	
**p65/RelA**	F	5´ ATGGCTTCTATGAGGCTGAGCT 3´	6FAM 5´ CATCCACAGTTTCCA 3´ MGB
	R	5´ CACACACTGGATTCCCAGGTT 3´	
**Musashi-2**	F	5´ AGATGTAAAGCAATATTTCGAGCAGTT 3´	6FAM 5´ TGGCAAGGTGGAAGAT 3´ MGB
	R	5´ TGGTAGTTTTATCAAACATCAGCATTG 3´	
**Hes1**	F	5´ GGACATTCTGGAAATGACAGTGAA 3´	6FAM 5´ ATGACGGCTGCGCTGA 3´ MGB
	R	5´ CAGCACACTTGGGTCTGTGC 3´	

Table S2. Median expression values of each of the genes analyzed

	Musashi-2	CYLD	Hes1	A20	EZH2	AEG-1	TRAF6	NFKBIA	p65/RelA	BRCA1	RAP80
**Median**	15.31	1.78	0.51	1.10	2.95	1.36	2.45	0.76	1.34	8.07	2.53
**Minimum**	1.22	0.60	0.06	0.26	0.49	0.29	0.65	0.20	0.19	0.86	0.48
**Maximum**	70.84	10.41	3.41	3.32	15.37	5.46	8.17	3.65	4.12	39.70	9.85
**No. patients***	59	58	53	57	39	57	57	60	60	55	59

*Discrepancies in patient numbers are due to technical issues related to the analyses.

Table S3. Correlation of the expression levels of the 11 genes analyzed

		BRCA1	RAP80	EZH2	AEG-1	NFKBIA	Musashi-2	p65/RelA	A20	CYLD	Hes1	TRAF6
**BRCA1**	**Pearson**	1										
	**p**											
**RAP80**	**Pearson**	0.139	1									
	**p**	0.312										
**EZH2**	**Pearson**	0.591	0.088	1								
	**p**	<0.001	0.596									
**AEG-1**	**Pearson**	0.510	0.133	0.168	1							
	**p**	<0.001	0.328	0.314								
**NFKBIA**	**Pearson**	0.111	0.038	0.109	0.543	1						
	**p**	0.422	0.774	0.507	<0.001							
**Musashi-2**	**Pearson**	0.520	0.252	0.383	0.352	0.258	1					
	**p**	<0.001	0.057	0.016	0.008	0.048						
**p65/Rel A**	**Pearson**	0.139	-0.035	0.080	0.465	0.590	0.387	1				
	**p**	0.311	0.795	0.628	<0.001	<0.001	0.002					
**A20**	**Pearson**	0.219	0.341	0.080	0.009	0.133	0.255	0.105	1			
	**p**	0.15	0.009	0.630	0.950	0.325	0.056	0.437				
**CYLD**	**Pearson**	0.353	0.505	0.361	0.167	0.012	0.444	-0.123	0.385	1		
	**p**	0.009	<0.001	0.026	0.218	0.929	0.001	0.357	0.003			
**Hes1**	**Pearson**	0.231	-0.108	0.396	0.199	0.272	0.374	0.379	0.085	0.027	1	
	**p**	0.103	0.441	0.015	0.158	0.049	0.006	0.005	0.544	0.851		
**TRAF6**	**Pearson**	0.502	0.326	0.334	0.310	-0.004	0.651	0.165	0.304	0.644	0.148	1
	**p**	<0.001	0.013	0.043	0.021	0.977	<0.001	0.221	0.024	<0.001	0.300	

Table S4. Gene expression levels according to the presence or absence of K-ras mutations

Gene	K-ras	N	Median	Minimum	Maximum	p
BRCA1	mutated	9	7.85222	3.970	15.330	0.34
	wild-type	42	9.85810	1.860	31.520	
	total	51	9.50412	1.860	31.520	
RAP80	mutated	10	2.9390	.86	5.69	0.76
	wild-type	45	3.1784	.48	9.85	
	total	55	3.1349	.48	9.85	
EZH2	mutated	9	3.8789	.49	10.80	0.77
	wild-type	28	3.5193	.88	15.37	
	total	37	3.6068	.49	15.37	
AEG-1	mutated	10	2.0626	.46	5.46	0.04
	wild-type	45	1.3443	.29	4.02	
	total	55	1.4749	.29	5.46	
NFKBIA	mutated	10	1.4797	.36	3.65	0.04
	wild-type	46	.9140	.20	3.13	
	total	56	1.0150	.20	3.65	
Musashi-2	mutated	10	17.0204	6.44	39.30	0.77
	wild-type	45	18.3834	1.22	70.84	
	total	55	18.1356	1.22	70.84	
p65/Rel A	mutated	10	1.6333	.62	3.64	0.47
	wild-type	46	1.4262	.19	4.12	
	total	56	1.4632	.19	4.12	
A20	mutated	10	1.0601	.33	2.62	0.57
	wild-type	44	1.1958	.26	3.32	
	total	54	1.1707	.26	3.32	
CYLD	mutated	10	2.3811	.60	6.23	0.77
	wild-type	45	2.5970	.67	10.41	
	total	55	2.5578	.60	10.41	
Hes1	mutated	8	.5234	.06	1.11	0.60
	wild-type	42	.6380	.07	3.41	
	total	50	.6197	.06	3.41	
TRAF6	mutated	10	2.3585	1.21	5.70	0.42

	wild-type	44	2.8318	.65	8.17	
	total	54	2.7442	.65	8.17	

Table S5. Gene expression levels according to the presence or absence of EGFR mutations (deletion in exon 19 or L858R in exon 21)

Gene	EGFR status	No	Median	Minimim	Maximum	p
BRCA1	mutated	8	9.49250	4.310	14.530	0.94
	wild-type	37	9.29946	1.860	39.700	
	total	45	9.33378	1.860	39.700	
RAP80	mutated	9	3.8511	1.01	9.50	0.13
	wild-type	40	2.7332	.48	9.85	
	total	49	2.9386	.48	9.85	
EZH2	mutated	4	2.2175	1.27	2.78	0.36
	wild-type	28	3.8439	.49	15.37	
	total	32	3.6406	.49	15.37	
AEG-1	mutated	8	1.0976	.29	2.01	0.27
	wild-type	40	1.5516	.41	5.46	
	total	48	1.4760	.29	5.46	
NFKBIA	mutated	9	.7055	.35	1.51	0.18
	wild-type	41	1.1157	.20	3.65	
	total	50	1.0418	.20	3.65	
Musashi-2	mutated	9	17.0823	3.72	37.96	0.99
	wild-type	40	17.0076	1.22	46.41	
	total	49	17.0213	1.22	46.41	
p65/Rel A	mutated	9	1.1627	.50	2.21	0.25
	wild-type	41	1.5052	.56	4.12	
	total	50	1.4436	.50	4.12	
A20	mutated	8	1.1366	.27	3.32	0.97
	wild-type	39	1.1265	.26	2.81	
	total	47	1.1282	.26	3.32	
CYLD	mutated	9	2.8297	1.05	6.32	0.45
	wild-type	39	2.2675	.60	10.41	
	total	48	2.3729	.60	10.41	
Hes1	mutated	8	.7169	.27	1.57	0.55
	wild-type	36	.5800	.06	3.41	
	total	44	.6049	.06	3.41	
TRAF6	mutated	8	2.7282	1.23	5.06	0.67

	wild-type	39	2.5025	.65	6.96	
	total	47	2.5409	.65	6.96	

Table S6. Correlation between gene expression levels and number of metastatic sites

	1 metastatic site N (%)	≥2 metastatic sites N (%)	p
BRCA1			0.09
Low	17 (68)	8 (32)	
High	10 (40)	15 (60)	
RAP80			0.99
Low	14 (54)	12 (46)	
High	15 (56)	12 (44)	
CYLD			0.78
Low	15 (58)	11 (42.3)	
High	13 (50)	13 (50)	
EZH2			0.49
Low	9 (53)	8 (47)	
High	6 (35)	11 (65)	
AEG-1			0.78
Low	14 (56)	11 (44)	
High	13 (50)	13 (50)	
NFKBIA			0.41
Low	17 (63)	10 (37)	
High	13 (48)	14 (52)	
Musashi-2			0.41
Low	16 (62)	10 (39)	
High	13 (48)	14 (52)	
Hes1			0.002
Low	19 (76)	6 (24)	
High	7 (29)	17 (71)	
TRAF6			0.99
Low	14 (56)	11 (44)	
High	14 (54)	12 (46)	
p65/RELA			0.17
Low	18 (67)	9 (33)	
High	12 (44)	15 (56)	
A20			0.78
Low	15 (56)	12 (44)	
High	12 (50)	12 (50)	

Table S7. Cox regression model for PFS including only gene expression levels

PFS	HR	95% CI	p
BRCA1	1.03	0.99-1.07	0.12
RAP80	0.95	0.83-1.09	0.48
EZH2	1.08	0.97-1.19	0.15
AEG-1	1.43	1.11-1.85	0.006
NFKBIA	1.20	0.83-1.74	0.34
Musashi-2	1.01	0.98-1.02	0.70
p65/RelA	1.23	0.85-1.79	0.27
A20	0.92	0.61-1.38	0.68
CYLD	0.95	0.81-1.11	0.54
Hes1	1.14	0.71-1.82	0.59
TRAF6	0.99	0.82-1.21	0.99
